# On Diseases of the Bladder and Prostate Gland

**Published:** 1843-01

**Authors:** 


Art. IX.—*On Diseases of the Bladder and Prostate Gland.* With Plates. By William Coulson. Third Edition, revised and corrected.—*London,* 1842. 8vo, pp. 274.

Having noticed the two former editions of this work, (see Vol. VI. p. 509, and Vol. X. p. 247,) we will now only say that it is still further improved in the present; particularly in the chapters devoted to the Urine, to Calculus, and to Affections of the Prostate. It is now a handsome volume, and one of great practical value.

